# Outcomes of liver diseases in pregnant females: A study from a tertiary care medical center in Pakistan

**DOI:** 10.12669/pjms.40.3.7670

**Published:** 2024

**Authors:** Nazish Butt, Sabir Ali, Haleema Yasmeen, Khalid Mumtaz

**Affiliations:** 1 Nazish Butt, FCPS. Gastroenterology Department, Jinnah Postgraduate Medical Centre, Karachi, Pakistan; 2 Sabir Ali, MBBS. Gastroenterology Department, Jinnah Postgraduate Medical Centre, Karachi, Pakistan; 3 Haleema Yasmeen, FCPS. Gastroenterology Department, Jinnah Postgraduate Medical Centre, Karachi, Pakistan; 4 Khalid Mumtaz, FCPS (Med), FCPS (GI), MSc Division of Gastroenterology, Hepatology & Nutrition, The Ohio State University, Columbus, United States

**Keywords:** Liver Diseases, Pregnancy, Etiology, HEV, HELLP, Fulminant Hepatic Failure, Maternal Complications, Fetal Complications, Maternal Mortality, Fetal Mortality

## Abstract

**Objective:**

To determine the etiologies and outcomes of liver disease in pregnancy in a developing country.

**Method:**

A total of 336 consecutive pregnant women with liver disease were included in this prospective cohort study conducted at the Department of Gastroenterology, Jinnah Postgraduate Medical Center, Karachi from August 2019 to August 2021. Patients’ baseline demographic, clinical, and laboratory data and outcomes were collected on a pre-designed questionnaire.

**Results:**

Among all the pregnant females, the most common liver disease was acute hepatitis E virus (HEV) infection (37.2%), followed by preeclampsia (PEC)/eclampsia (EC), hemolysis, elevated liver enzymes & low platelets (HELLP) syndrome, and hyperemesis gravidarum (HG). The most common maternal complications were fulminant hepatic failure (FHF) in 14.9% and placental abruption in 11.0%. Fetal complications included intrauterine death (IUD) in 20.8% and preterm birth in 8.6%. The maternal and neonatal mortality rates were 11.6% and 39.6%, respectively. Among the predictors, low maternal weight, low body mass index (BMI), and low hemoglobin (Hb) were associated with increased maternal mortality. Low fetal weight, height, maternal systolic blood pressure (SBP), and low maternal Hb were independent predictors of fetal mortality.

**Conclusion:**

In our cohort of pregnant females in a tertiary care medical center, acute HEV was the most common liver disease, followed by PEC/EC, HELLP, and HG. Maternal and fetal deaths were alarming in this group of patients and demanded careful management.

## INTRODUCTION

Liver diseases are rare but can result in potentially serious complications of pregnancy. The prevalence of liver disease during pregnancy in Western countries is approximately 3%, resulting in morbidity and mortality in both mother and fetus.^1^ Liver diseases during pregnancy are classified into two main categories; those related to pregnancy and those that are non-related or coincidental to pregnancy.^1^ Non-pregnancy-related liver diseases can occur at any time of pregnancy, however, pregnancy-related liver disorders manifest at specific trimesters. For instance, HG, a pregnancy-related liver disorder, exhibits clinical manifestations in early pregnancy. In contrast, others include Intrahepatic Cholestasis of Pregnancy (ICP), preeclampsia (PEC) with liver involvement including HELLP syndrome, and acute fatty liver of pregnancy (AFLP) affect later in late pregnancy.^1^ It is crucial to note the onset of clinical symptoms and abnormal liver function tests (LFTs) for appropriate and timely diagnosis and management.

The prevalence of these pregnancy-related liver conditions varies greatly; it has been observed that HG complicates approximately 0.3-2.0% of pregnancies during the 1^st^ trimester.^1^ ICP incidence rate displays geographic variations, i.e., 0.5% to 1.8% of pregnancies are known to be affected by ICP in Europe. In comparison, the highest peaks are reported from the South American countries and Scandinavia (28%).^1^,^2^-^5^ While HELLP syndrome is a rare variant of severe PEC, around 12% of these patients suffer from HELLP syndrome.^6^ Another rare condition, AFLP complicating the third trimester, affects one in every 16,000 pregnancies.^7^

The pregnancy-related outcomes are primarily affected by the severity of the liver disease rather than the disease etiology.^1^ It is highly recommended to use the prognostic scoring systems to ease the prediction of pre-pregnancy maternal decompensation risk.^8^ Variceal bleeding is reported to lead to maternal mortality among pregnant patients with underlying cirrhosis and portal hypertension.^8^ The deterioration continues with pregnancy and peaks in the second trimester; the increased blood volume in the circulation and exertion of direct pressure on the inferior vena cava by the gravid uterus impairs venous return. There is a 25% risk of variceal hemorrhagic episodes during pregnancy among patients with pre-existent varices.^1^ The literature also confirms a mortality rate of up to 25% among pregnant females with liver diseases.^9^

We aimed to determine etiologies, clinical features, and outcomes related to liver disease in pregnancy. The rationale for conducting this research was to fill in these knowledge gaps, enhance the capability of the medical community to identify, manage, and give early interventions for pregnant women with liver illnesses by examining these conditions extensively. By expanding the understanding and practices linked to liver problems in pregnancy, this study will help improve the patient care, reducing the maternal and fetal morbidity and mortality.

## METHODS

This prospective cohort study included 336 consecutive pregnant women with all types of liver diseases either specific to pregnancy and nonspecific to pregnancy presented at the Department of Gastroenterology, Jinnah Postgraduate Medical Center, from August 2019 to August 2021.

### Ethical Approval

The study protocol was approved by the Ethical Review Committee of JPMC (No. F.2-81/2019-GENL/041/JPMC). All ethical guidelines were followed, and data confidentiality was maintained.

The sample size of 222 cases was calculated using expected percentage of liver disease as 5.6%^10^, 95% confidence interval and 7% margin of error. All pregnant females with liver disease were included in this study regardless of gravida and parity, while pregnant females with advanced renal disease and underlying malignancy were excluded.

The primary endpoint was to assess the fetal and maternal outcomes in terms of survival and death. While secondary endpoints included the assessment of predictors affecting these outcomes. The data was collected using a pre-designed questionnaire, including age, ethnicity, weight, height, BMI, education level, and residence. Laboratory data were collected on the biochemical tests, including Hb, urea, total bilirubin (TB), creatinine, prothrombin time and international normalized ratio (PT & INR), activated partial prothrombin time (APPT), albumin, alanine aminotransferases (ALT), aspartate aminotransferases (AST), and alkaline phosphatase (ALP).

Frequencies and percentages were reported for qualitative variables like education level, residence, etiological factors, and outcome. Mean with standard deviation was calculated for all quantitative variables like age, weight, height, BMI, hemoglobin, and other laboratory tests. The effect modifiers were controlled through stratification of age, education, residence, and etiologies to observe their effect on outcome variables by applying the Chi-square test. Furthermore, a multivariable logistic regression model was used to estimate maternal and fetal mortality predictors. A p-value < 0.05 was considered statistically significant, and SPSS version 18.0 was used to analyze the data.

## RESULTS

During the study, 336 pregnant females with liver disease were identified at the JPMC. The mean age of pregnant women was 27.50 ± 6.04 years. The baseline demographic, clinical, and obstetric characteristics of the patients are shown in Table-I.

**Table-I:** Baseline demographic, clinical, and obstetric characteristics of the patients.

Variable		Mean ± SD
Age (years)		27.50±6.04
Weight (kg)		65.16±8.29
Height (m)		157.72±6.64
BMI (kg/m^2^)		34.46±157.04
SBP (mmHg)		118.83±21.00
DBP (mmHg)		76.90±17.10
Hb (g/dl)		10.06±1.85
		*n (%)*
Ethnicity	Sindhi	172 (51.2)
	Urdu Speaking	114 (33.9)
	Pashtoon	17 (5.1)
	Punjabi	14 (4.2)
	Balochi	10 (3.0)
	Bangali	9 (2.7)
Maternal Education	Illiterate/Non-Primary	167 (49.7)
	Primary	56 (16.7)
	Secondary	68 (20.2)
	Higher secondary	45 (13.4)
Residence	Urban	222 (66.1)
	Rural	114 (33.9)
Comorbidities	None	247 (73.5)
	HTN	77 (22.9)
	CLD	12 (3.6)
Addiction	None	295 (87.8)
	Mawa	6 (1.8)
	Betel nut chewer	35 (10.4)
Pregnancy	Primigravida	52 (15.5)
	Multigravida	284 (84.5)
Gestational Age	2nd trimester	44 (13.1)
	3rd trimester	273 (81.3)
	Not Reported	19 (5.7)

HTN-Hypertension; CLD- Chronic Liver Disease; TB-Total Bilirubin; PT- Prothrombin Time; INR- international normalized ratio; APTT- activated partial thromboplastin time; Alb-Albumin; ALT- Alanine Aminotransferase; AST- aspartate aminotransferase; ALP-Alkaline phosphatase

Nausea (44.6%), was the most frequently reported clinical presentation followed by jaundice (42.9%) and vomiting (31.0%). Among liver diseases nonspecific to pregnancy, the most common was acute HEV infection (37.2%), followed by acute HCV (25.6%), acute HBV (7.1%). PEC/EC (10.4%) was the most common among diseases specific to pregnancy, followed by AFLP (7.7%). The study observed a maternal mortality rate of 11.6% and reported fetal deaths in 39.6% of cases. Notable complications included FHF in 14.9% of cases and placental abruption in 11.0% of cases. Fetal complications were marked by IUD in 20.8% and preterm birth in 8.6% of cases (Table-II).

**Table-II:** Clinical presentations, management complications, and outcomes of liver disease in pregnancy.

Variable					N(%)
Clinical presentations		Fever			48 (14.3)
		Jaundice		144 (42.9)	
		Nausea		150 (44.6)	
		Vomiting		104 (31.0)	
		RUQ pain		42 (12.5)	
		Abdominal pain		16 (4.8)	
		Pruritus		4 (1.2)	
		ALOC		121 (36.0)	
		Cirrhosis		18 (5.4)	
		CTP	Class A	18 (5.4)	
			Not Applicable	318 (94.6)	
Etiologies of liver disease Specific to Pregnancy			Nonspecific to Pregnancy (Hepatitis)	Acute HEV	125 (37.2)
			Acute HCV	86 (25.6)	
			Acute HBV	24 (7.1)	
			Chronic HBV	6 (1.8)	
			PEC/EC	35 (10.4)	
			HELLP	22 (6.5)	
			HG	9 (2.7)	
			AFLP	26 (7.7)	
			ICP	3 (0.9)	
Treatment			Anti-Emetic		9 (2.7)
		Anti-Hypertensive		16 (4.76)	
		Sof+Dec		86 (25.6)	
		Supportive		151 (44.9)	
		Symptomatic		44 (13.1)	
		Tenofovir		30 (8.9)	
Outcome		Maternal	Survived		297 (88.4)
			Death		39 (11.6)
		Fetal	Survived		203 (60.4)
			Death		133 (39.6)
Complications		Maternal	FHF		50 (14.9)
			Placental abruption		37 (11.0)
		Fetal	IUD		70 (20.8)
			Preterm		29 (8.6)

RUQ- Right Upper Quadrant; ALOC- Altered Level of Consciousness; CTP- Child-Turcotte-Pugh; FHF- fulminant hepatic failure; IUD- intrauterine death; PEC/EC - Preeclampsia/Eclampsia; HELLP - hemolysis, elevated liver enzymes, and low platelets; HG-Hyperemesis Gravidum; AFLP - Acute Fatty Liver Of Pregnancy; ICP -Intrahepatic Cholestasis Of Pregnancy

The patterns of the biochemical laboratory values among pregnant females with various pregnancy-specific and nonspecific diseases is summarized in Table-III. The highest ALT levels were found in patients with acute hepatitis E virus infection, followed by AFLP and HELLP, while AST was highest among ICP patients, followed by acute hepatitis E and HELLP.

**Table-III:** Clinical characteristics with respect to Liver Disease among Pregnant females.

Disease									

Variable	Specific To Pregnancy			Others		Nonspecific To Pregnancy (Hepatitis)			

	HG	PEC/EC	HELLP Syndrome	ICOP	AFLP	Acute HBV	Acute HCV	Acute HEV	Chronic HBV
Hb	5.70±0.00	10.07±2.47	10.76±2.73	9.30±0.00	9.72±0.50	10.40±0.31	10.86±1.21	9.68±0.77	11.00±0.00
Urea	18.00±0.00	31.45±9.74	42.40±10.14	74.00±0.00	55.19±56.98	20.91±8.30	30.52±11.37	72.87±71.63	30.00±0.00
TB	1.09±0.00	1.01±0.72	4.71±5.46	8.88±0.00	13.34±6.14	0.66±0.39	0.62±0.30	14.12±7.08	0.20±0.00
Cr	0.54±0.24	0.83±0.28	1.57±0.54	3.30±0.00	1.37±0.40	0.58±0.18	0.87±0.20	1.51±0.44	1.00±0.00
PT	9.60±0.00	15.18±6.21	11.90±1.31	11.40±0.00	24.30±7.16	10.58±0.50	12.91±2.08	21.26±4.42	12.00±0.00
INR	0.88±0.00	1.2814±0.47	1.06±0.10	1.07±0.00	2.04±0.55	0.95±0.05	1.14±0.13	1.82±0.32	1.00±0.00
APPT	20.00±0.00	29.54±0.85	28.18±2.10	33.00±0.00	31.07±2.33	28.91±0.28	29.76±0.87	30.79±3.49	30.0±0.00
ALB	3.90±0.00	3.67±0.57	3.07±0.80	2.91±0.00	3.78±0.25	4.05±0.09	3.62±0.47	3.45±0.57	3.46±0.00
ALT	18.00±0.00	94.00±157.18	178.68±80.61	432.00±0.00	1368.96±1141.7	15.75±4.23	44.08±18.78	2059.52±2199.98	13.00±0.00
AST	40.00±0.00	77.22±112.31	140.45±90.11	793.00±0.00	135.76±39.57	17.79±4.25	26.60±5.77	480.66±803.50	29.00±0.00
ALP	556.00±0.00	532.45±354.16	815.18±110.60	1211.33±7.50	610.30±116.68	311.16±201.66	516.83±236.68	897.03±519.38	235.00±0.00

Among the baseline demographic and obstetric characteristics, pregnancy, gestational age, education, ethnicity, residence, and types of pregnancy-specific and nonspecific etiologies of liver disease were found to be significantly associated with maternal outcomes (survival/death) (p<0.05). Like maternal outcomes, fetal deaths were also most common among those affected with acute HEV (49.6%).

Independent predictors of maternal and fetal outcomes are summarized in Table-IV. Low weight, low BMI, and low Hb, higher urea, creatinine, total bilirubin, higher INR were maternal mortality predictors. Similarly, fetal weight & height, maternal SBP & low Hb were predictors of fetal mortality along with higher urea, creatinine, total bilirubin, INR, and APTT.

**Table-IV:** Independent Predictors of Maternal and fetal mortality.

Variables	Maternal mortality		p-value	Fetal mortality		p-value
	
	OR	95% CI		OR	95% CI	
Age	1.04	0.98-1.10	0.156	0.99	0.95-0.70	0.705
Weight	1.05	1.00-1.10	0.019*	1.06	1.03-0.00	<0.001*
Height	0.95	0.90-1.00	0.068	1.03	1.00-0.04	0.046*
BMI	1.22	1.07-1.39	0.002*	0.99	0.99-0.45	0.458
SBP	1.01	0.99-1.02	0.182	1.02	1.00-0.00	0.001*
DBP	0.99	0.97-1.01	0.836	1.00	0.99-0.19	0.197
Hb	1.38	1.15-1.67	0.001*	1.18	1.05-0.00	0.005*
Urea	0.98	0.97-0.98	<0.001*	0.98	0.97-0.00	<0.001*
Creatinine	0.20	0.10-0.37	<0.001*	0.12	0.07-0.00	<0.001*
TB	0.85	0.81-0.89	<0.001*	0.95	0.93-0.00	0.001*
PT	0.84	0.79-0.89	<0.001*	0.92	0.88-0.00	<0.001*
INR	0.09	0.04-0.20	<0.001*	0.35	0.22-0.00	<0.001*
APTT	0.93	0.84-1.03	0.180	0.88	0.81-0.00	0.007*

* p<0.05 is considered statistically significant.

## DISCUSSION

The study conducted in Karachi, Pakistan, found higher rates of complications and maternal and fetal mortality in pregnant females with liver diseases. The maternal and fetal mortality rates were 11.6% and 39.6%, respectively. However, previous studies from smaller and remote centers in Southeast Asia reported even higher rates of maternal and neonatal mortality.^11^-^13^ Brohi et al., reported 28.8% and 77%, maternal and fetal mortality, respectively.^11^ Similarly, Rathi et al., reported 19.7% and 35.4%, respectively.^12^ A local study from Pakistan reported six maternal deaths; two due to hepatitis E, one each from HELLP and AFLP, and two remained undiagnosed. The overall perinatal mortality within the group was 43%.^14^ The higher mortality rates may be attributed to the fact that these are older studies from smaller centers.

Unlike other studies, encephalopathy, disseminated intravascular coagulation (DIC), liver hematoma or intra-abdominal bleeding, etc., were not frequent in the present study. Acute HEV was associated with FHF in all cases, HCV was linked to 83.3% of placental abruptions, and HELLP Syndrome was found in 16.2% of cases. Maternal mortality rates were high in HEV-related FHF, and there were high rates of intrauterine deaths (IUDs) in pregnancies complicated by HEV. These findings are consistent with a previous study reporting high IUD rates in pregnant women with hepatitis and PEC.^15^

Our results suggest that the most common cause of acute liver failure was acute HEV. This is supported by a recent systematic review that reported a higher prevalence of HEV infection among symptomatic pregnant females.^16^ A local seroprevalence study reported that 15.6% of pregnant females (symptomatic) were suffering from HEV infection.^17^ Similarly, an Indian study including symptomatic pregnant females reported that the HEV prevalence was 41.9%.^18^ HEV infection during pregnancy increases the risk of fetal and maternal mortality, possibly due to hormonal imbalances and suppression of the immune system.^16^,^19^

Surprisingly, HCV infection accounted for 25.6% of the cases of liver diseases in the present study. In one study, it accounted for a significant percentage of liver disease cases, while in another study, it was relatively low and had no adverse effect on pregnancy outcomes.^20^ Similar results have been reported in other studies, with some reporting a higher prevalence and others reporting a lower prevalence.^21^-^23^

We found variable patterns of liver function tests in patients with different diagnoses. The highest ALT levels were found in acute HEV patients, followed by AFLP and HELLP. While AST was highest among ICP patients, followed by acute Hepatitis E and HELLP. Furthermore, abnormal total bilirubin was observed among patients with acute Hepatitis E, AFLP, ICP, and HELLP. A similar pattern of LFTs was reported by Bigna et al. among pregnant women with HELLP and AFLP.^16^ Gaba et al., reported abnormal LFTs among 60.3% women with HG.^24^

Lastly, concerning the predictors of maternal and fetal mortality, we found that maternal weight, BMI, and hemoglobin predicted increased maternal mortality, whereas maternal height, SBP, weight, and Hb were among the predictors of fetal mortality as per the logistic regression analysis. Another study by Meng et al. assessed maternal and fetal mortality risk factors among pregnant females with acute fatty liver; they reported an increased risk of maternal mortality among females with prolonged PT, nausea, and raised creatinine levels.^25^

Our study adds valuable insights to the existing medical literature regarding liver diseases among pregnant females. The study revealed essential data on complications, maternal and fetal mortality, as well as the frequency of liver diseases. It contributes to the understanding of the role of HEV in adverse pregnancy outcomes, possibly related to hormonal imbalances and immune system suppression.

### Limitations

Our study was conducted at a single center and had certain limitations. Some important data points were missing in our patients, such as data related to serum bile levels in patients with ICP. Secondly, our sample size was small, and therefore some of the results need to be interpreted carefully. However, despite these few limitations, we managed to report important findings in pregnant patients.

## CONCLUSION

Acute HEV was the most common etiological factor nonspecific to pregnancy, while PEC/EC, HELLP, and HG were pregnancy-specific etiologies of liver disease. Women with acute HEV, HCV, and HELLP Syndrome displayed the highest frequency of maternal and perinatal complications. Lastly, the study reported significant maternal & fetal mortality rates and identified their predictors. There is a need to work on modifiable predictors identified in our study and improve maternal and fetal mortality and morbidity.

### Authors Contribution:

**NB**, **HY** and **SA:** Conception and design, acquisition of data, analysis and interpretation of data.

**NB** and **KM:** Drafting the article and revising it critically for important intellectual content.

**NB** and **HY:** Final approval of the version to be published.

All authors are in agreement to be accountable for all aspects of the work in ensuring that questions related to the accuracy or integrity of any part of the work are appropriately investigated and resolved.
